# Functional characterization of 42 CK2α *de novo* variants associated with Okur‐Chung neurodevelopmental syndrome

**DOI:** 10.1111/febs.70538

**Published:** 2026-04-13

**Authors:** Alexander Gast, Christian Werner, Karsten Niefind, Joachim Jose

**Affiliations:** ^1^ Institute for Pharmaceutical and Medicinal Chemistry University of Münster Germany; ^2^ Department of Chemistry and Biochemistry, Institute of Biochemistry University of Cologne Germany

**Keywords:** CK2, CSNK2A1, neurodevelopmental disorders, OCNDS, protein kinase

## Abstract

In 2016, *CSNK2A1*, the gene which encodes the catalytic α‐subunit of human protein kinase CK2 was linked to an autism spectrum disorder called Okur‐Chung neurodevelopmental syndrome (OCNDS) for the first time. Human protein kinase CK2 is a heterotetrameric phosphotransferase with an α_2_β_2_ composition. To gain more insight into genotype–phenotype relationships, 42 CK2α variants associated with OCNDS were characterized in this study in terms of enzymatic activity using a canonical CK2 peptide substrate. Out of the 42 variants tested, 13 had no detectable enzymatic activity and 12 showed less than 10% of wild‐type CK2α activity. The addition of the regulatory CK2β subunit increased the activity of all active variants. Twelve variants that exhibited at least 30% wild‐type enzymatic activity were chosen to determine the dissociation constants with CK2β; highlighting that none of these mutations had an impact on the interaction of CK2α with CK2β. The variants R21Q, T127M, E264D, E282K, and R333* showed no reduced activity in comparison to wild‐type CK2α. Additionally, affinity to CK2β and thermostability remained unaltered. K198R is the most frequent missense variant observed in OCNDS patients; thus, we performed a site‐saturation mutagenesis at position K198 to elucidate what impact other variants at this position could have on enzymatic activity. Results showed that replacement of K198 by any other amino acid resulted in similar loss of activity as the prevalent K198R variant. The presented results suggest that other parameters beyond enzymatic activity, CK2β affinity or thermostability may contribute to neurodevelopmental disorders such as OCNDS.

## Graphical abstract

Okur‐Chung neurodevelopmental syndrome (OCNDS) is a neurodevelopmental disorder associated with mutations in the gene coding for Protein kinase CK2α. In this work, 42 variants of CK2α associated with OCNDS were characterized *in vitro*. This included determination of catalytic activity and CK2α/CK2β‐interaction as well as an assessment of evolutionary conservation. Variants with apparently unaltered biochemical function *in vitro* were purified and structurally characterized in‐depth.

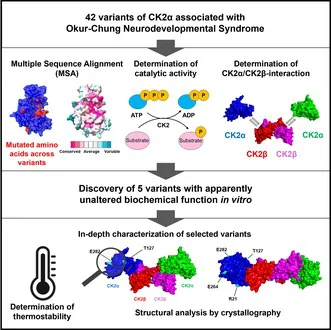

**Abbreviations**
CK2: protein kinase CK2—formerly “casein kinase 2”
MSA: multiple sequence alignment
NDD: neurodevelopmental disorder
OCNDS: Okur‐Chung neurodevelopmental syndrome
SSM: site‐saturated mutagenesis
WT: wild‐type CK2α

## Introduction

Protein kinase CK2 is a constitutively active enzyme forming tetrameric holoenzymes consisting of two catalytic subunits CK2α or CK2α′ and two so‐called regulatory subunits CK2β [1]. CK2, present in all eukaryotic organisms, is highly conserved throughout the phylogenetic tree and is ubiquitously expressed in all tissues [2]. The minimum substrate consensus sequence of CK2 has been described as S/T‐x‐x‐D/E/pS/pT, a motif reflected by around 30% of phosphosites in human proteins [3]. Therefore, it is not surprising that hundreds of CK2 substrates have been identified so far [4, 5]. Moreover, affinity of substrates to CK2 is increased by further acidic residues surrounding the phosphorylation site (P), most prominently at the P + 1 position, leading to a consensus sequence described as S/T‐D/E‐x‐D/E [4, 6]. Due to its pleiotropic nature, CK2 is involved in various biological processes including embryonic development, cell cycle regulation, circadian rhythms, DNA damage response, and apoptosis [5, 7, 8, 9, 10, 11]. Knockout of CK2α or CK2β has been shown to be embryonically lethal in mice [12, 13], while knockout of CK2α' is leading to infertility of male mice [14]. It is worth mentioning that only vertebrates seem to exhibit the CK2α′ isoform [15]. Unsurprisingly, its pleiotropy makes CK2 a target in many diseases, such as COVID‐19, autoimmune disorders and a variety of cancer types [16, 17, 18, 19]. CK2 is highly expressed in the brain [9, 20] and has been related to a variety of neurodegenerative diseases such as Alzheimer's disease and Parkinson's disease [16, 21, 22]. In particular CK2α has been described as a modulator of receptor endocytosis and neurotransmitter signaling within the brain [20, 23, 24]. Heterozygous mutations of the genes *CSNK2A1* and *CSNK2B* coding for CK2α or CK2β respectively, have been associated with the neurodevelopmental disorders (NDDs) Okur‐Chung neurodevelopmental syndrome (OCNDS) [25] and Poirier‐Bienvenu neurodevelopmental syndrome (POBINDS) [26]. NDDs are among the most prevalent diseases affecting more than 3% of children worldwide [27, 28]. Due to their role in neuronal development and function, a variety of protein kinases have been associated with NDDs in the past [29]. The association of CK2 with both neurodegenerative and neurodevelopmental disorders suggests a crucial role in brain development and function. OCNDS is characterized by a broad spectrum of symptoms which have recently been reviewed by Bagatelas *et al*. (2025). Some common clinical features include mild to severe autism spectrum disorder, intellectual disability, language impairment, attention deficit hyperactivity disorder, microcephaly, and craniofacial disorders. In all cases, some form of developmental delay has been observed [25, 30, 31, 32, 33, 34, 35, 36, 37, 38, 39, 40]. OCNDS was first discovered by exome sequence analysis of patients exhibiting such symptoms, which allowed relation to mutations within *CSNK2A1* [25]. To our knowledge, 123 different mutations have been described to date either in scientific literature or in databases such as the registry of the CSNK2A1 foundation (https://www.csnk2a1foundation.org/registry‐dashboard (retrieved 20th of May, 2025)) [25, 30, 31, 32, 33, 34, 35, 36, 37, 38, 39, 40]. These comprise 86 missense, 22 frame shift and 15 nonsense mutations. Fascinatingly, different mutations ranging from missense mutations in the interdomain to extremely truncated variants lead to a similar overarching phenotype. These mutations appeared to be heterozygous dominant, which means that they lead to the described symptoms despite the presence of a second wild‐type allele. However, it has been reported that two patients carrying the same mutation can differ strongly in terms of symptom burden [41].

More recently, multiple *in vitro* studies have been performed to characterize variants associated with OCNDS [42, 43, 44, 45]. All variants associated with OCNDS tested so far showed a reduced enzymatic activity with CK2 consensus substrates. The most prevalent variant K198R was shown to also affect substrate specificity [42, 44]. The objective of this study was to expand the knowledge on the features of so far non‐characterized variants. For this purpose, 41 missense variants and one nonsense variant were analyzed in terms of enzymatic activity with a canonical CK2 peptide substrate. Additionally, selected variants were characterized for CK2α/CK2β interaction and thermal stability, as well as by crystal structure analysis.

## Results

### Enzymatic activity of the selected OCNDS CK2α‐variants

In total, 42 variants of CK2α related to Okur‐Chung neurodevelopmental syndrome (OCNDS) were selected for this work to cover all known functional domains (Table 1). The selection of the variants has been rationalized in the materials and methods section.

**Table 1:** Variants of CK2α associated with OCNDS investigated in this work.

Variant	Number of patients^a^	Functional domain^b^	Source^c^
N16I	1	N‐terminal segment	CSNK2A1‐foundation registry
R21Q	1	N‐terminal segment	Jafari Khamirani *et al*. [36], Unni *et al*. [38]
E27K	3	N‐terminal segment	Lelieveld *et al*. [46], Chiu *et al*. [30]
Y39C	1	N‐terminal segment, CK2β‐binding	Wang *et al*. [47]
Y39S	1	N‐terminal segment, CK2β‐binding	Unni *et al*. [38]
G46V	1	ATP‐binding, Glycine‐rich loop, CK2β‐binding	Unni *et al*. [38]
G48S	3	ATP‐binding, Glycine‐rich loop, CK2β‐binding	CSNK2A1‐foundation registry
Y50N	1	ATP‐binding, Glycine‐rich loop, CK2β‐binding	CSNK2A1‐foundation registry
E52K	1	CK2β‐binding	Unni *et al*. [38]
V53L	1	CK2β‐binding	Unni *et al*. [38]
V66L	1	ATP‐binding CK2β‐binding	CSNK2A1‐foundation registry
L70P	1	CK2β‐binding	CSNK2A1‐foundation registry
L70R	1	CK2β‐binding	CSNK2A1‐foundation registry
V73E	1	CK2β‐binding	Chiu *et al*. [30]
T127M	1	Hinge plus αD	Wang *et al*. [47]
C147Y	1	Interdomain	Wang *et al*. [47]
M153R	1	Catalytic loop	Unni *et al*. [38]
I174M	1	ATP‐binding	Owen *et al*. [31]
G177S	4	Mg^2+^‐binding loop, Activation segment	Unni *et al*. [38]
L178W	1	Activation segment, Activation loop	Unni *et al*. [38]
E180K	2	Activation Segment, Activation loop	CSNK2A1‐foundation registry
R191P	3	Activation segment, P + 1 loop	CSNK2A1‐foundation registry
A193P	2	Activation segment, P + 1 loop	CSNK2A1‐foundation registry
S194F	5	Activation segment, P + 1 loop	Unni *et al*. [38], CSNK2A1‐foundation registry
S194P	1	Activation segment, P + 1 loop	CSNK2A1‐foundation registry
R195P	1	Activation segment, P + 1 loop	CSNK2A1‐foundation registry
R195Q	2	Activation segment, P + 1 loop	CSNK2A1‐foundation registry
Y196C	1	Activation segment, P + 1 loop	CSNK2A1‐foundation registry
F197I	1	Activation segment, P + 1 loop	Owen *et al*. [31]
K198R	81	Activation segment, P + 1 loop	CSNK2A1‐foundation registry, Unni *et al*. [38]
K198T	1	Activation segment, P + 1 loop	Zhao *et al*. [37]
G199D	1	Activation segment, P + 1 loop	Unni *et al*. [38]
D214V	1	Interdomain	CSNK2A1‐foundation registry
S217N	4	Interdomain	CSNK2A1‐foundation registry
A223E	1	Interdomain	CSNK2A1‐foundation registry
P231R	1	Interdomain	Lelieveld *et al*. [46], Chiu *et al*. [30]
Q241E	2	Interdomain	CSNK2A1‐foundation registry
E264D	1	Interdomain	CSNK2A1‐foundation registry
E282K	1	Interdomain	CSNK2A1‐foundation registry
R312Q	3	Interdomain	Chiu *et al*. [30], Unni *et al*. [38]
R312W	8	Interdomain	Owen *et al*. [31], CSNK2A1‐foundation registry
R333*	1	Interdomain	Unni *et al*. [38]

^a^ Number of patients gives the highest value observed either in the patient registry or in scientific literature.

^b^ Classification into functional domains according to *Unni et al*. [38].

^c^ CSNK2A1‐foundation registry can be accessed at https://www.csnk2a1foundation.org/registry‐dashboard (as accessed on 20.05.2025).

CK2 enzymatic activity of these variants was determined as described before [45]. For this purpose, *Escherichia coli* cells expressing the respective CK2α^1–335^ variant as a fusion protein with the red fluorescent protein mScarlet (mScar) were used [48]. Briefly, cells were lysed after protein expression had been induced, and the concentration of CK2α was determined by the red fluorescence of the fusion protein in the lysates. CK2α^1–335^ was used, as full‐length CK2α is prone to degradation at the C terminus [49] and may lead to involuntary release of the C‐terminally fused mScar domain. CK2α^1–335^ was shown to be functionally similar to full‐length CK2α in regards to enzymatic activity and CK2α/CK2β‐interaction and therefore serves as an appropriate model *in vitro* [50, 51]. Hence, CK2α^1–335^ is referred to as wild type (WT) in this work. As substrate for the activity measurements, the synthetic peptide RRRDDDSDDD was used, which contains the CK2 consensus sequence. It has been used in a number of studies before, making it generally considered as a canonical CK2 substrate [52, 53, 54, 55]. CK2 enzymatic activity in each lysate was determined with a capillary electrophoresis (CE)‐based activity assay [56]. Phosphorylation of the substrate peptide leads to a change in electrophoretic mobility, allowing the separation of substrate and product by CE. Substrate and product can be quantified by CE‐UV detector at 195 nm.

The results of the activity measurements of the CK2α variants are shown in Fig. 1A. The kinetic data for activity measurements is attached in Table S1. Out of the 42 variants tested, 37 showed impaired enzymatic activity. Among these, 13 had no measurable activity at all (Y39C, Y39S, G46V, C147Y, M153R, A193P, S194F, S194P, G199D, D214V, A223E, R312Q, R312W) and for 12 further variants, the enzymatic activity was reduced to less than 10% of WT level. Eight variants had an activity between 10 and 30% and four variants had an activity between 30 and 80% of WT. To our surprise, five variants (R21Q, T127M, E264D, E282K, and R333*) exhibited no significantly reduced enzymatic activity compared to WT (one‐way ANOVA, *P* < 0.01).

**Fig. 1:**
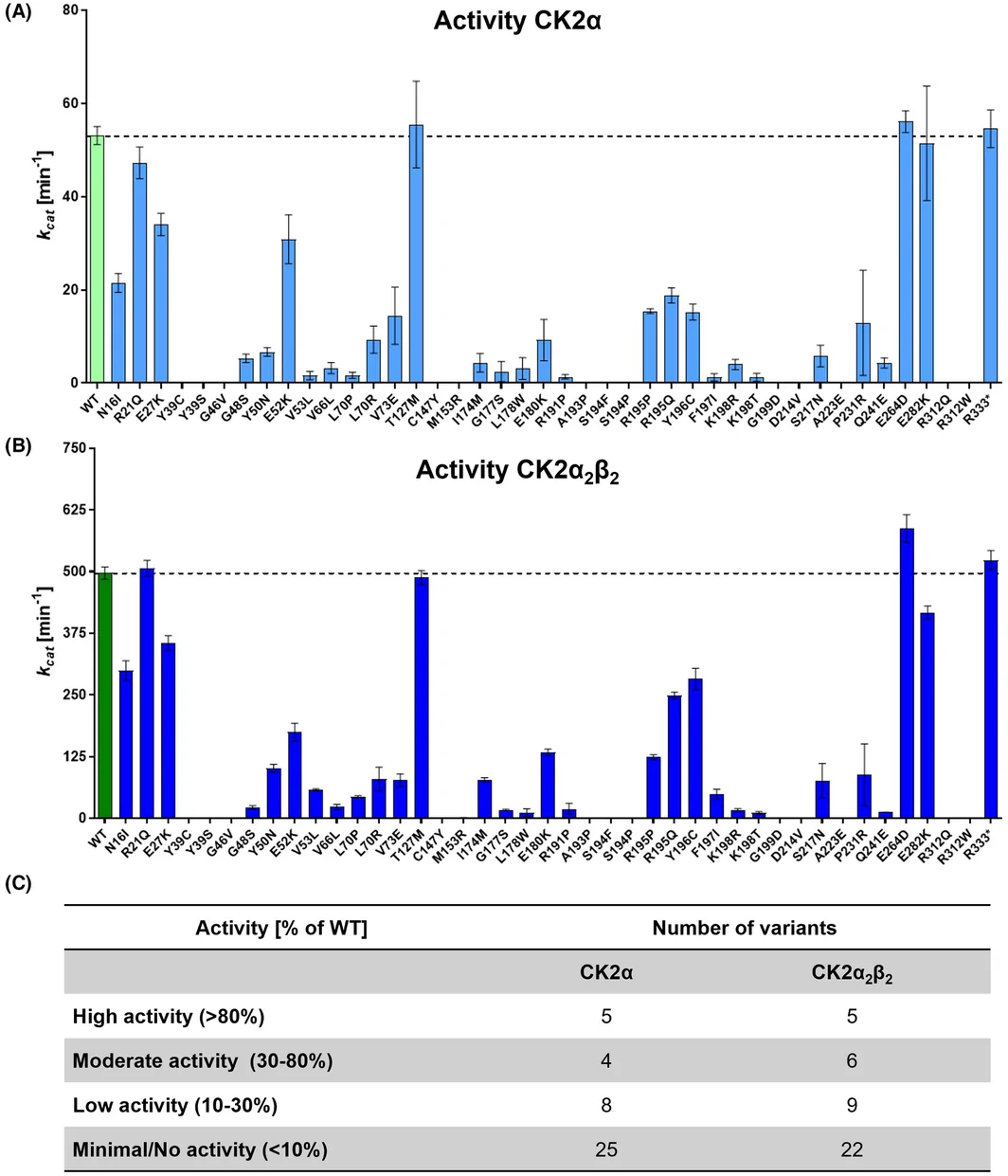
Activity determination of CK2α variants in *Escherichia coli* cell lysates. Concentrations of the variants were set to be 10 nm. Activities were determined without addition of CK2β (A) and in presence of an excess of 200 nm CK2β (B). The dotted line indicates the activity of the WT. (C) Summary of variant activity levels. Data are presented as mean ± SD (*n* = 3 biological replicates).

### Addition of CK2β increases enzymatic activity for all active variants

For measuring holoenzyme (CK2α_2_β_2_) activity, a twenty‐fold excess of CK2β^1–193^ that was purified before [44] was added to the cell lysate. The results of the activity measurements with the holoenzyme are shown in Fig. 1B. A truncated variant CK2β^1–193^ was used instead of full‐length CK2β because it is less prone to aggregation, while maintaining full functionality [50]. It will be referred to as CK2β for better readability. The results as obtained with CK2α and the holoenzyme CK2α2β2 with the different variants are summarized in Fig. 1C.

Although the enzymatic activity of the CK2α variants substantially differed, the addition of CK2β led to an increase in activity for all, except those without any kinase activity. WT showed around 9‐fold increase in enzymatic activity upon addition of CK2β. Most variants with moderate (30–80%) or high activity (> 80%) had a similar increase in activity after addition of CK2β. For the variants N16I, E180K, R195Q, and Y196C, enzymatic activity was increased 13–18‐fold, indicating that the formation of the holoenzyme partly compensated the effect of the mutation. Yet, none of the variants was recovered to full activity after addition of CK2β. E52K variant exhibited only a 5.6‐fold increase in activity upon addition of CK2β, indicating that the substitution of glutamine at position 52 by lysine has a higher impact on the holoenzyme than on the catalytic subunit alone. After addition of CK2β, E282K showed a slightly yet significantly (one‐way ANOVA, *P* < 0.01) reduced activity (85%) compared to WT and the variant E264D showed a slightly (one‐way ANOVA, *P* < 0.01) increased activity (115%) compared to WT.

### Interaction of CK2α variants with CK2β

The interaction of CK2α variants toward CK2β was analyzed by a method described recently [45]. In this approach, the dose‐dependent activity enhancement of CK2α by addition of CK2β is used as a classical binding isotherm to determine dissociation constants (*K*_*D*_‐values). The *K*_*D*_‐values of the 12 most active CK2α variants with CK2β were determined. These comprised the five variants with nonreduced enzymatic activity in comparison to WT and seven additional variants exhibiting 30–80% activity of the WT holoenzyme. As CK2 activity was the readout of the assay, the dynamic range of the assay was severely reduced for variants with low activity. Hence, variants with an activity below 30% compared to WT were excluded from this experiment. Results are given in Fig. 2. As a control, the *K*_*D*_‐value of WT with CK2β was determined and turned out to be 8.6 nm, which was identical with the values described in previous reports [44, 45, 57]. The *K*_*D*_‐values of the variants selected varied within 4.9 nm to 13.6 nm. However, when the confidence intervals of these *K*_D_‐values were determined by Monte–Carlo simulations [45], it turned out that for all variants, these confidence intervals of the *K*_*D*_‐values overlapped with the one of WT CK2α. In consequence, it needs to be noted that the *K*_*D*_‐values of the variants and that of WT were not significantly different.

**Fig. 2:**
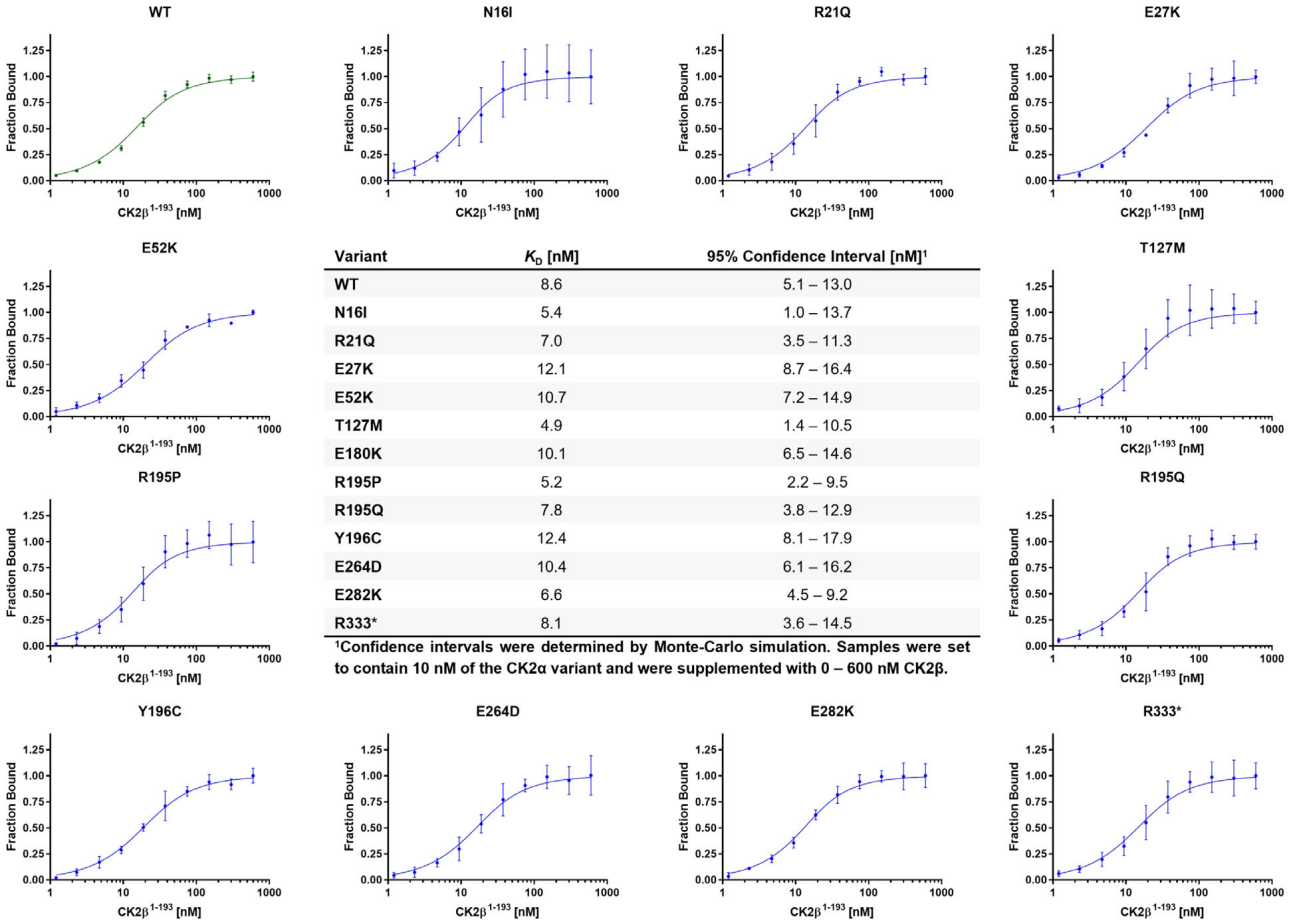
Dissociation constants of CK2α variants with the regulatory subunit CK2β, including experimentally determined binding isotherms. Data are presented as mean ± SD (*n* = 3 biological replicates).

### Thermostability of the CK2α variants with an unaltered enzymatic activity

The variants R21Q, T127M, E264D, and E282K appeared unaltered in comparison to WT in terms of activity and CK2α/CK2β‐interaction. To underlay these unexpected results, these variants except stop variant R333*, which seemed too similar to CK2α^1–335^, applied as WT here, were purified without a fusion to mScar. The enzymatic activity of the purified variants as determined with and without CK2β are shown in Fig. 3A,B. None of the variants showed a heavily impaired enzymatic activity compared to WT, with or without CK2β. Only T127M displayed a slight, but significantly reduced activity of about 80% in both enzyme forms. This was in contrast to the results with T127M in cell lysates. To find a possible reason for this discrepancy, the thermostability of the same variants, R21Q, T127M, E264D, and E282K was determined by differential scanning fluorimetry (DSF) as described previously [44], with and without CK2β. As shown in Fig. 3C WT and variants T127M, E264D, and E282K showed a melting point of around 43 °C, whereas variant R21Q had a significantly reduced melting point of 39.2 °C. After addition of CK2β the thermostability of all variants was increased (Fig. 3D). Nevertheless, R21Q exhibited a lower melting point of 53.6 °C compared to melting points of around 56 °C for WT and all other variants. These results indicate that for screening purposes, the cell lysate assay appears suitable, but the results, at least for some variants can require confirmation with purified enzymes.

**Fig. 3:**
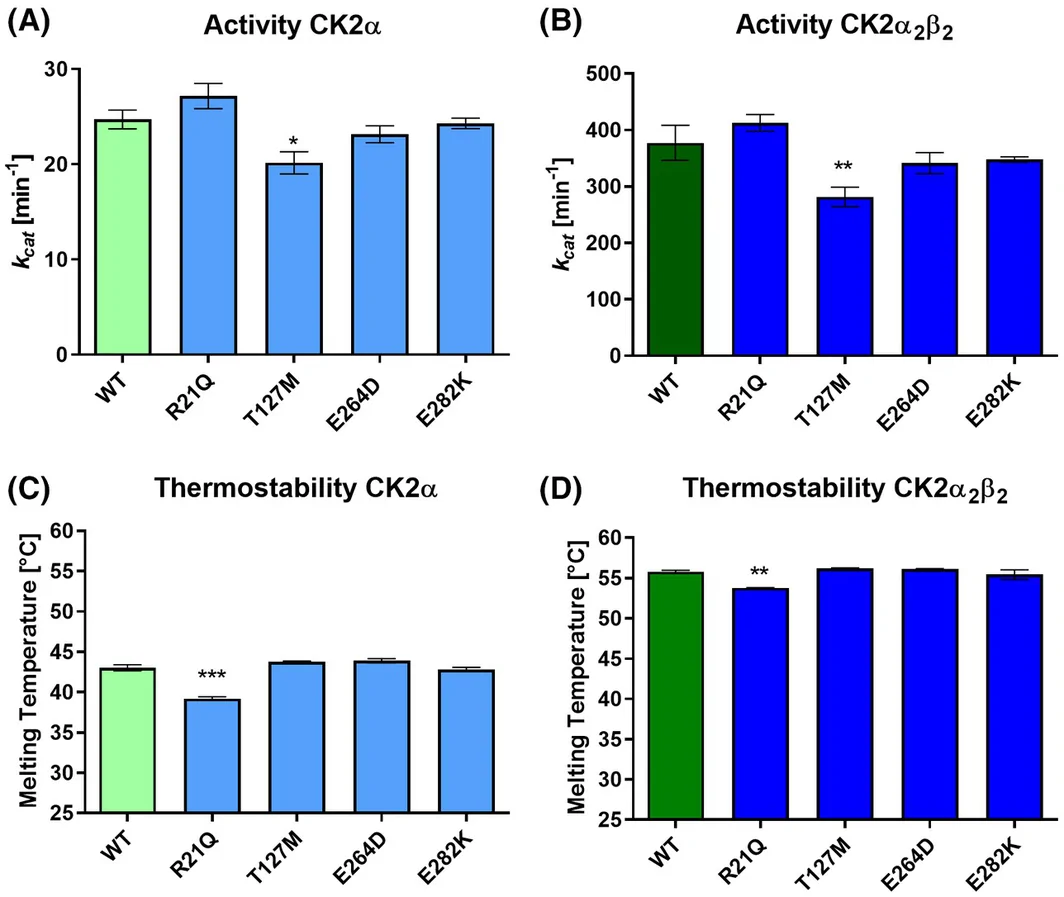
Enzyme activity and thermostability of purified variants R21Q, T127M, E264D, and E282K. Enzymatic activity of CK2α variants without addition of CK2β (A) and with addition of CK2β (B). Data are presented as mean ± SD (3 biological replicates). Thermostability analysis by Differential Scanning Fluorimetry (DSF) of CK2α variants without addition of CK2β (C) and with addition of CK2β (D). Data are presented as mean ± SD (*n* = 3 biological replicates in technical duplicates). Significance was calculated by one‐way ANOVA with WT set as control. *P < 0.05, **P < 0.01, ***P < 0.001.

### Crystal structure studies of the CK2α variants with an unaltered enzymatic activity

As shown in Fig. 4A,B, all mutations as present in the variants with an unaltered or only slightly reduced enzymatic activity are located at the surface of CK2α. They appeared neither involved in obvious intramolecular interactions, nor are they located close to known α/β interaction sites. In order to find out, whether these variants were structurally different to WT, the purified variants were subjected to crystal structure analysis as described previously [44].

**Fig. 4:**
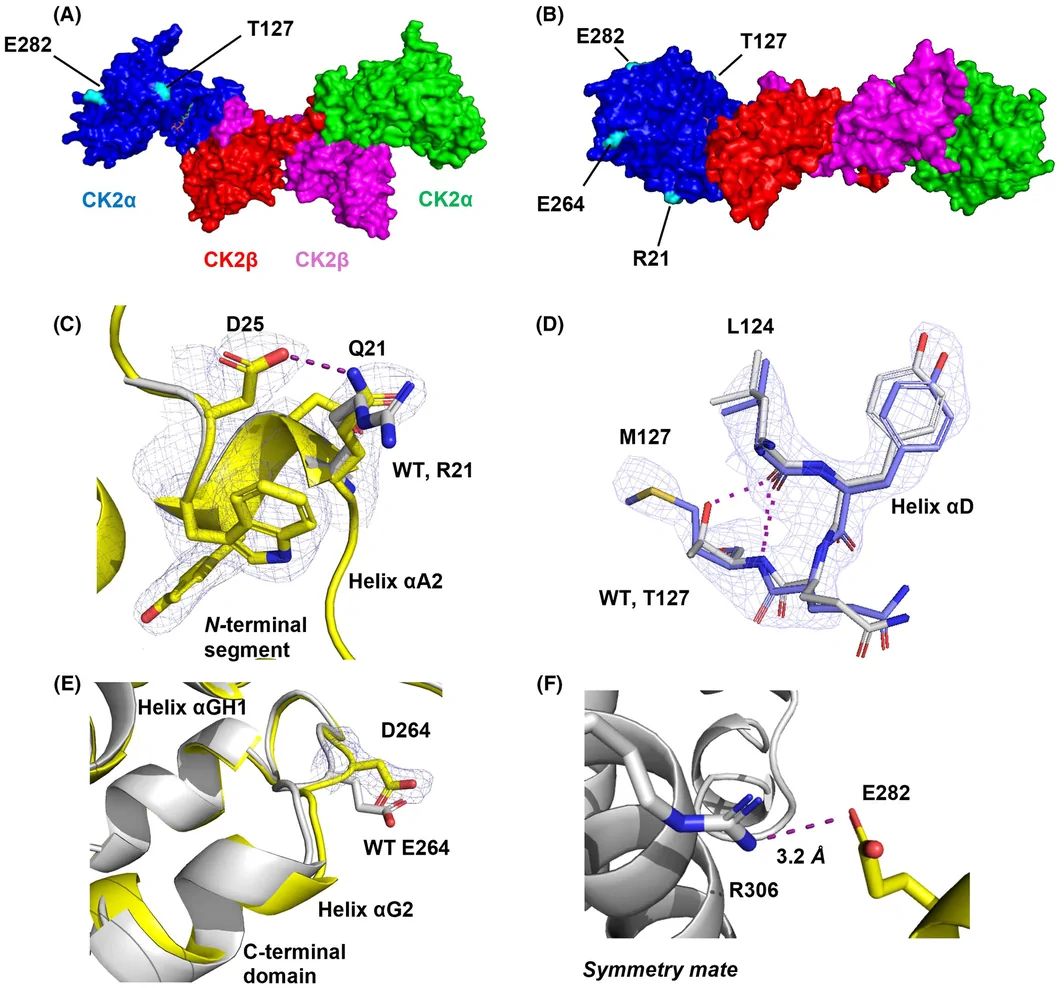
Front (A) and bottom (B) view of the CK2α_2_β_2_‐holoenzyme (PDB: 1JWH). The amino acids R21, T127, E264 and E282 are highlighted. Snapshots from the R21Q (PDB:9RCY) (C), T127M (PDB:9RCX) (D) and E264D (PDB:9RFN) (E) crystal structures, as well as representative crystal contact in the WT structure (PDB:2PVR), which hampered E282K crystallization attempts (F). R21Q causes an additional hydrogen bond in the N‐terminal segment. T127M causes the loss of a hydrogen bond within helix αD. The E264D crystal structure is ambiguous to us. A crystal contact mediated by E282 (here representatively taken from the R21Q crystal structure shown in (A)) likely prevented a successful crystallization of mutant E282K. The electron density is displayed at a Sigma cut‐off of 1.0. Structures of R21Q (C) and E264D (E) are shown in yellow, T127M (D) is shown in blue and WT structures are shown in gray in all compartments for better comparison. The figures were made with Pymol (Schroedinger).

X‐ray diffraction data and refinement statistics are shown in Table 2. Crystal structures were obtained for R21Q (PDB: 9RCY), T127M (9RCX), and E264D (9RFN) and showed high similarity to the WT structures. For this reason, only differences are highlighted in the following.

**Table 2:** X‐ray diffraction data and refinement statistics of CK2α mutant crystals.

Complex	CK2α^R21Q^	CK2α^T127M^	CK2α^E264D^
PDB code	9RCY	9RCX	9RFN
Wavelength	0.96770	0.87313	0.87313
Synchrotron (beamline)	MASSIF3, (ID30A‐3), ESRF	ID30B, ESRF	ID30B, ESRF
Space group	P 43 21 2	P 43 21 2	P 43 21 2
a, b, c [Å]	129.751129.751124.957	128.099128.099124.285	128.408128.408124.512
α, β, γ [°]	90.0 90.0 90.0	90.0 90.0 90.0	90.0 90.0 90.0
Resolution [Å] (highest shell)	91.748–2.355 (2.755–2.355)	73.202–2.253 (2.481–2.253)	89.389–2.577 (2.621–2.577)
*R*_sym_ [%]^a^	38.4 (250.6)	31.7 (222.7)	56.7 (308.3)
CC1/2^a^	0.975 (0.432)	0.997 (0.698)	0.991 (0.381)
Signal‐to‐noise ratio (I/*σ*_I_)^a^	6.4 (1.6)	9.4 (1.5)	7.1 (1.5)
No. of unique reflections^a^	24 764 (1238)	34 073 (1704)	31 814 (1658)
Completeness (spherical) [%]^a^	55.4 (7.5)	69.0 (14.0)	95.0 (100.0)
Completeness (ellips.) [%]^a^, ^b^	91.6 (73.7)	91.1 (73.0)	n. a.
Multiplicity^a^	6.5 (10.4)	25.6 (23.3)	26.0 (28.1)
Wilson‐plot B‐factor [Å^2^]	35.87	28.82	19.36
No. of refl. for *R*_work_/*R*_free_	24 749/2007	34 019/2005	31 575/2615
*R*_work_/*R*_free_ [%]	20.90/26.08	20.46/24.50	24.07/27.99
Protomers per asymmetr. unit	2	2	2
No. of non‐H‐atoms	5712	5883	5836
Protein	5566	5583	5594
Ligand/ion	60	50	64
Water	86	250	178
Average B‐Factors [Å^2^]	37.86	36.67	43.65
Protein	37.68	36.53	43.58
Ligand/ion	68.12	69.08	75.83
Water	28.49	33.21	34.29
R. m. s. d.			
Bond lengths [Å]	0.002	0.002	0.002
Bond angles [°]	0.41	0.49	0.49
Ramachandran plot			
Favored [%]	96.48	96.03	97.56
Allowed [%]	3.37	3.82	2.29
Outliers [%]	0.15	0.15	0.15

^a^ The values in brackets refer to the highest resolution shell.

^b^ After anisotropic analysis with STARANISO.

As depicted in Fig. 4C, an additional hydrogen bond in helix αA1 between Q21 and D25 was identified for variant R21Q. The T127M exchange results in a loss of a hydrogen bond in the helix αD originally formed between the sidechain of T127 and L124 (Fig. 4D). For variants E264D (Fig. 4E), the loop between helix αGH1 and αGH2 was shifted, bringing the carboxylic acid group of D264 slightly to the front, matching roughly with that of E264 in the wild‐type structure. Finally, we were not able to crystallize variant E282K. In WT crystals, E282 is located in an axis of symmetry and forms a salt bridge with R306 of an adjacent CK2α molecule (Fig. 4F). In E282K, this interaction is lost, most likely preventing crystal formation.

### Multiple sequence alignment

To gain further insight what impact R21, T127, E264, and E282 could have on the function of CK2α, a multiple sequence alignment (MSA) of human CK2α with its isoforms in 150 selected organisms was performed, using the Consurf server [58, 59]. As a result, conservation scores were generated for each amino acid in human CK2α. Unsurprisingly, most of the highly conserved amino acids were located in functional loop domains surrounding the catalytic cleft (Fig. 5A,B). A notable exception is R312, which is located close to the C‐terminal domain, but also showed a strong conservation. The impact of R312 is most probably due to a salt bridge formed with E201, which appears to stabilize the C‐terminal segment of CK2α [38]. The enzymatic activity of tested variants was overlayed with the conservation score of the amino acid at the corresponding position (Fig. 5A,C). As seen in Fig. 5C, there was a strong correlation (*r* = 0.8479) between catalytic activity and conservation score meaning that variations at positions that appeared to be highly conserved had the highest negative impact on enzymatic activity. The least conserved amino acids in CK2α were mostly located outside of key kinase domains, in regions such as the N‐ and C‐terminal segment (amino acids 1–39 and 330–391, respectively). Remarkably, in the CK2α variants related to OCNDS none of these amino acids appeared to be altered. R21, T127, E264, and E282 were located at positions with a degree of low conservation, corresponding to the most variable amino acid positions found to be altered in OCNDS. This is in accordance with the unaltered high enzymatic activity of R21Q, T127M, E264D, and E282K, the most active out of all tested variants.

**Fig. 5:**
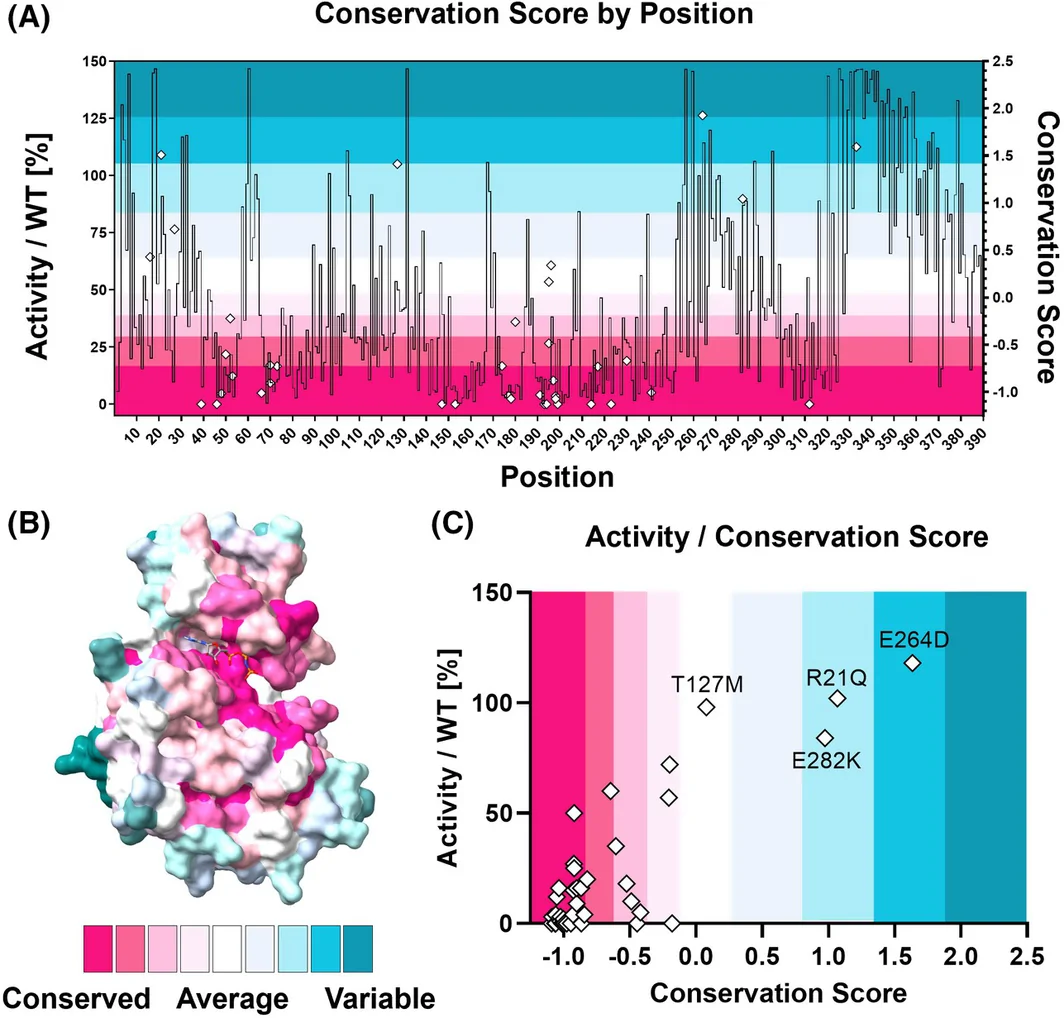
Conservation of amino acids in human CK2α. (A) shows the conservation score of all amino acids as calculated by the Consurf server software. A conservation score of −1.0 represents a highly conserved amino acid, while a conservation score of 2.5 represents a highly variable amino acid. The activity of the CK2α_2_β_2_ holoenzyme of OCNDS‐related variants tested in this work is given as ◇. (B) Structure of CK2α (PDB:2PVR) with amino acids colored according to their conservation score. Protein structure was presented using the software UCSF ChimeraX. (C) Visualization of the activity of CK2α‐variants found in OCNDS in relation to the conservation score of the respective mutated amino acid. Raw data is provided in Figures S1 and S2.

The raw data of the MSA are provided in Figures S1, S2. It showed that Q appeared in place of R21 in 10% of the analyzed organisms. Organisms containing the variant R21Q in CK2α include a variety of yeast strains such as *Saccharomyces cerevisiae*, but also invertebrates, such as *Hyalella azteca*, an amphipod crustacean. However, CK2α in these organisms has many further variations in comparison to human CK2α, in contrast to patient variants, which contain only a single amino acid exchange each. Nonetheless, the existence of R21Q in these organisms could be an indication for what reason the R21Q mutation has only limited effects on enzymatic activity, thermostability, and α/β interaction. Although appearing less frequently, similar observations were made for T127M (2% of all organisms analyzed), E264D (5%), and E282K (4%).

### Sequence alignment of CK2α with CK2α'

When human CK2α was compared with human CK2α′ in an amino acid sequence alignment using MegAlign Pro (DNASTAR, Madison, WI, USA) (Fig. 6), it turned out that despite an overall sequence identity of 85%, the amino acids at position 127 and 264 were different. Position T127 was represented by I128 in CK2α′ and position E264 was represented by D265 in CK2α′. For E264D, this was exactly the same missense variation as appearing in OCNDS patients. This means that a missense variation leading to OCNDS in CK2α is naturally occurring in CK2α′. It appears remarkable and could be explained by either other mutations in CK2α′ overcoming the effect of the amino acid exchange E to D, or an effect of D265 in CK2α′ does not lead to similar effects as the corresponding D264 in CK2α due to different expression patterns.

**Fig. 6:**
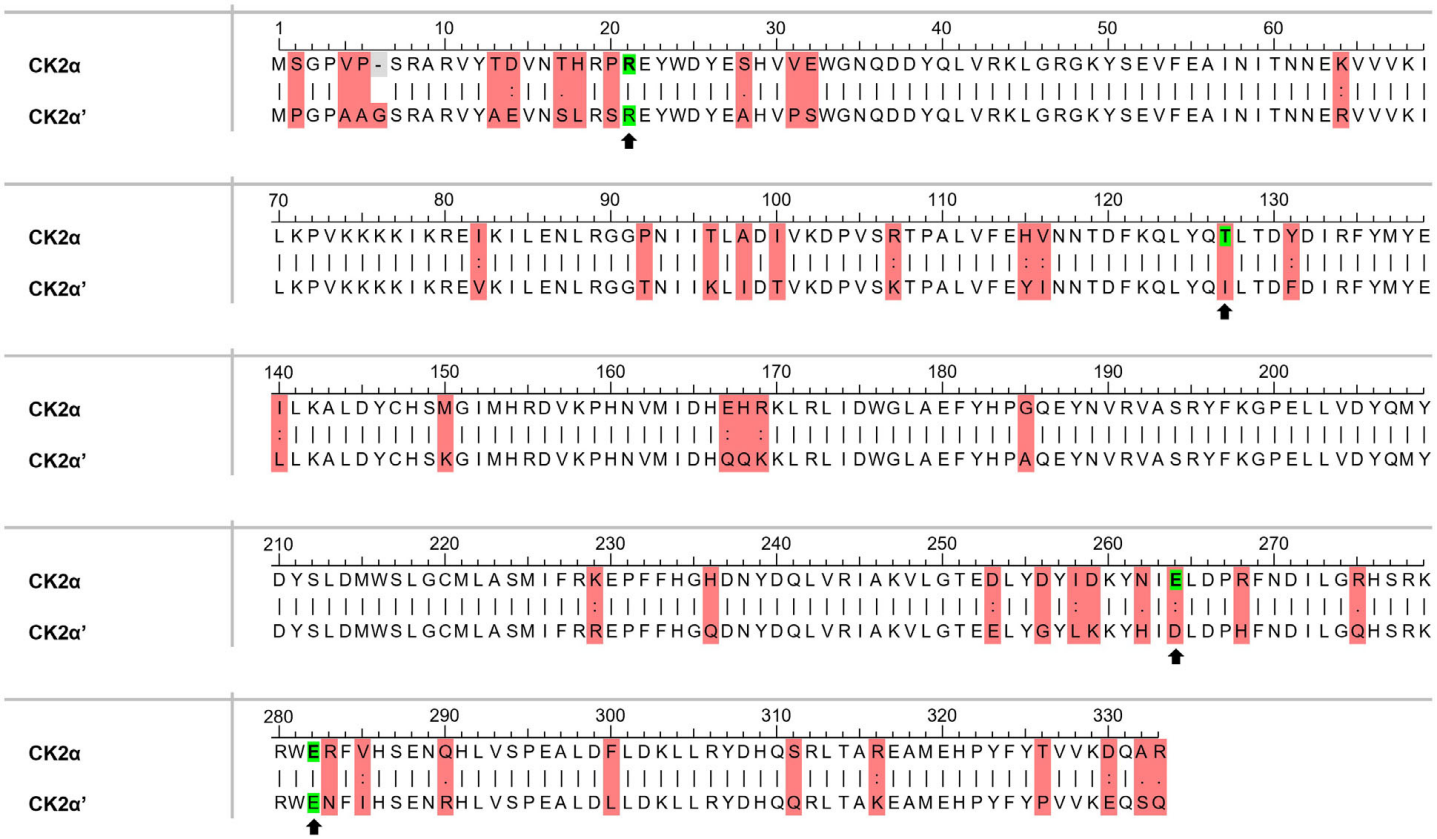
Sequence alignment of human CK2α and CK2α'. Varying amino acids are highlighted in red. The amino acids R21, T127, E264 and E282 are labeled with an arrow and highlighted in green. Sequence alignment was performed using MegAlignPro 17 (DNASTAR).

Similar to what was observed for human CK2α′, CK2α analogues in other organisms, for example, model organisms *Drosophila melanogaster* (dmCK2α) and *Caenorhabditis elegans* (ceCK2α) also contain the E264D variation. An MSA of CK2α, CK2α′, dmCK2α, and ceCK2α is shown in Fig. 7.

**Fig. 7:**
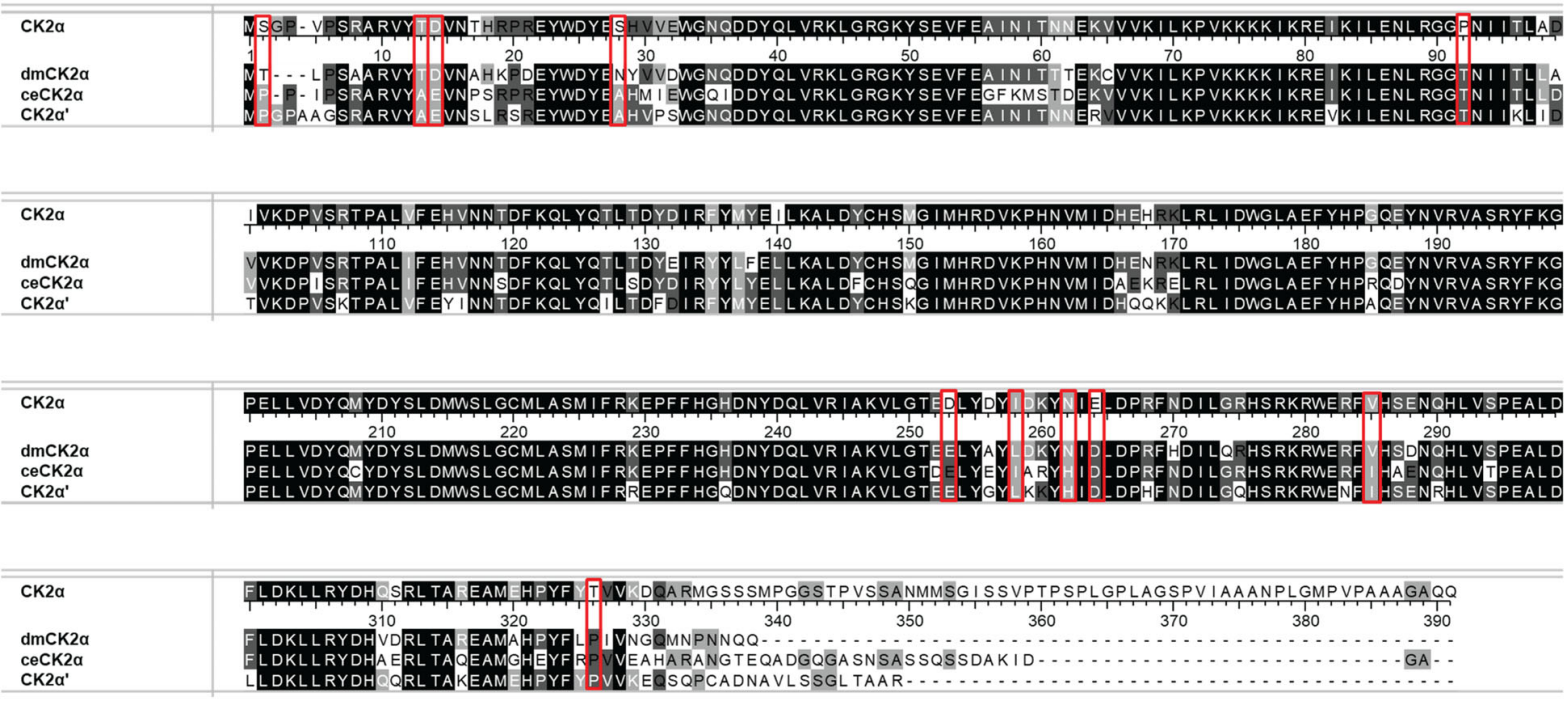
Multiple sequence alignment (MSA) of human CK2α and CK2α' with CK2α from *Drosophila melanogaster* (dmCK2α) and *Caenorhabditis elegans* (ceCK2α). Residues which are identical in CK2α' and dmCK2α or in CK2α' and ceCK2α, but different in CK2α, are highlighted in red. Sequence alignment was performed using MegAlignPro 17 (DNASTAR).

The MSA revealed that several amino acid residues are shared between dmCK2α, ceCK2α, and human CK2α′, but not with human CK2α. Many of these common residues are located in or around the helix αGH1 and αGH2 (D253 (CK2α′: E254), I258 (L259), N262 (H263), and E264 (D265)). Figure 8A–C show that both helices αGH1 and αGH2 are far away from any known functional domains. An overlay of CK2α and CK2α′ (Fig. 8B) shows that many of the amino acids differing between the isoforms are facing towards the protein surface. Additionally, the surface of the helix αGH1 contains more acidic residues in CK2α compared to CK2α′. This charge difference should make CK2α more attractive for positively charged interaction partners in this region. As a result, the sequence variations could contribute to isoform‐specific substrate recognition in human CK2α.

**Fig. 8:**
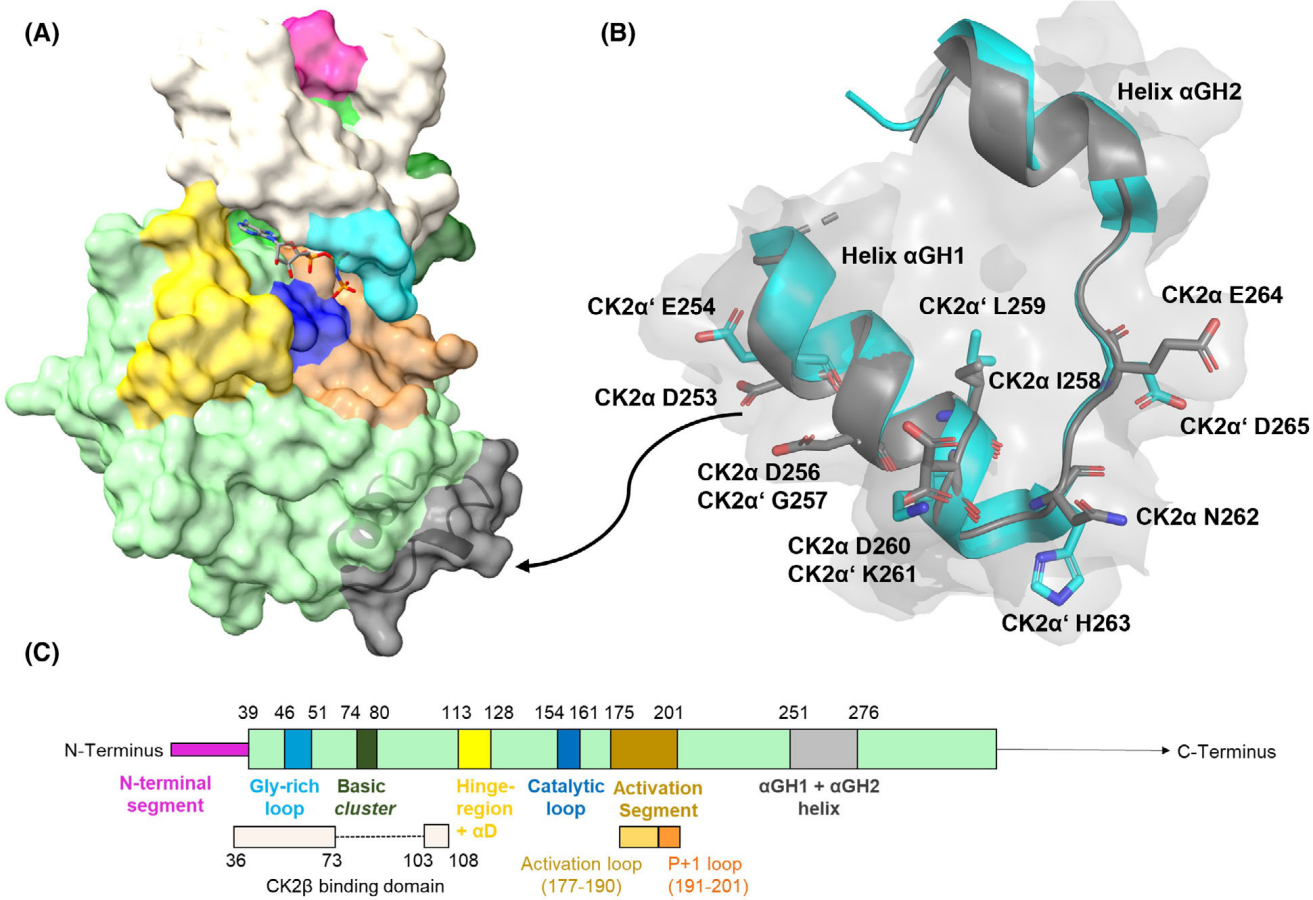
Structure of helix αGH1 and αGH2 of CK2α. Frontal view of the CK2α subunit (A) shows the location of the helix αGH1 and αGH2 in gray within CK2α. Alignment of CK2α in gray (PDB:2PVR) and CK2α' in turquoise (PDB:6TGU) (B) in the area of helix αGH1 and αGH2. Amino acids differing between the isoforms are named and numbered. Functional domains are colored according to the respective color in the protein overview (C). Protein structures were presented using the software UCSF ChimeraX.

Notably, *D. melanogaster* and *C. elegans* both lack a gene for the isoform CK2α′ [60, 61, 62]. The sequence identities between human CK2α and CK2α′ toward dmCK2α (88.7%/81.7%) and ceCK2α (78.6%/79.2%) could be an indication for a hybrid CK2α/CK2α′ in these organisms. It indeed has recently been discussed that CK2α′ is evolutionarily more ancient, with CK2α in vertebrates representing the rather recent adaptation [15]. Further investigation of structural differences of vertebrate CK2α, including its altered helix αGH1, might be the key to better understand the distinct functions of CK2α and CK2α′ [63].

### Site‐saturated mutagenesis at position K198 of human CK2α

As of now, 81 OCNDS patients have been reported to possess the K198R variant and only a single patient was reported to carry the K198T variant, whereas other substitutions at this position have not appeared [37, 38]. To see whether enzymatic activity lays a selective pressure on this amino acid position, a site‐saturated mutagenesis (SSM) was performed. For this purpose, the codon for K198 was randomized, using degenerate primers to generate a library encoding all 20 possible amino acids at position 198. A total of 72 different *Escherichia coli* BL21(DE3) colonies obtained after transformation, each containing a single K198X variant, were selected for analysis. By such a sample size, the library was supposed to statistically represent 18.7 out of the 20 possible amino acids. Cells were cultivated and lysed as above and the CK2α concentration in each lysate was determined by fluorescence measurement of the fusion proteins as described before [45]. Finally, the lysates were supplemented with an excess of 200 nm CK2β for CK2 holoenzyme enzymatic activity determinations. It was shown that with the exception of two variants, all other variants showed an activity below 5% of WT levels (Fig. 9A). The two variants with an extraordinarily high activity, as well as nine variants with low activity were subjected to Sanger sequencing. It turned out that the two variants with the highest activity contained the WT codon K198. The other variants were identified as K198Q (3×), K198R (1×), K198L (2×), K198T (2×), and K198F (1×). Despite hardly detectable differences in activity for the bulk of variants, K198Q appeared to be the most active out of them. Therefore, K198Q, WT and the OCNDS‐associated variants K198R and K198T were again cultivated and the enzymatic activity was determined for each variant in six biological replicates (Fig. 9B). The enzymatic activities of K198R, K198T, and K198Q were similarly low at around a level of 5% of WT, with no statistically significant difference between them.

**Fig. 9:**
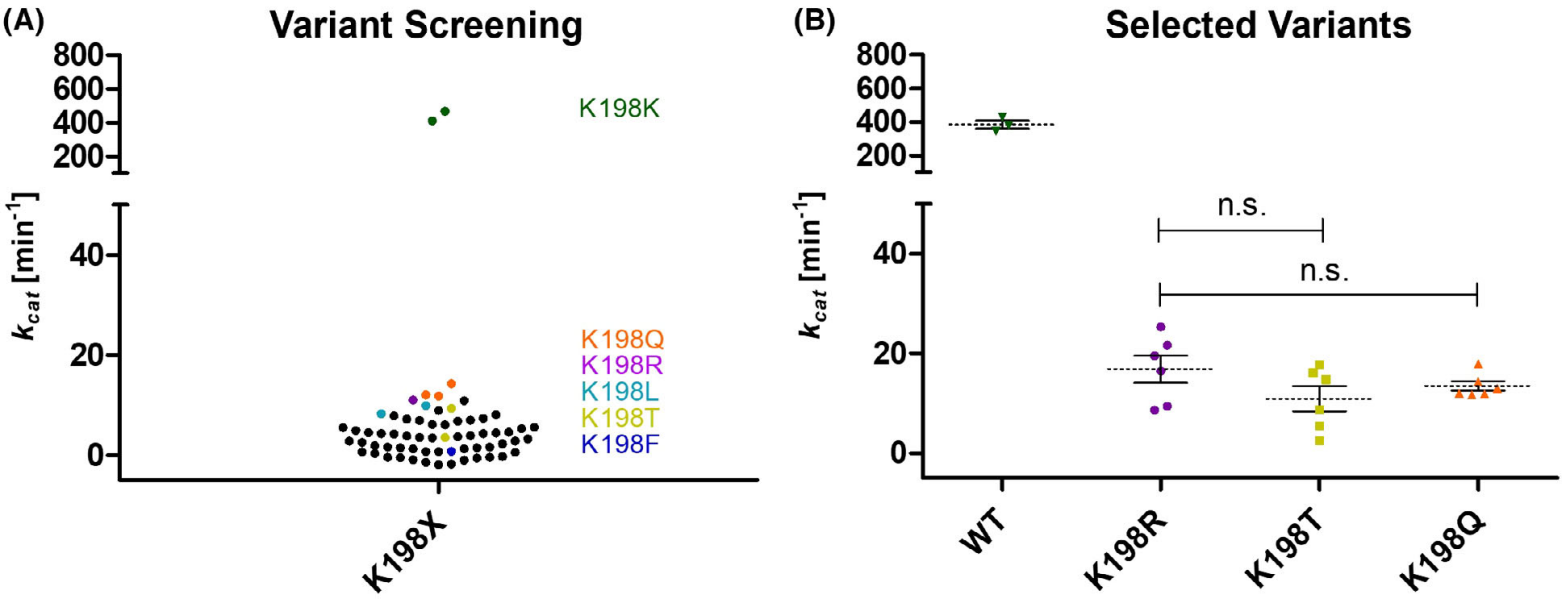
Results of site saturation mutagenesis of Lys198 in activity assay. A plasmid library with a degenerated codon for Lys198 was generated and transformed in *Escherichia coli* BL21(DE3). Unique colonies were chosen to generate cell lysates containing unique variants of CK2α^K198X^. (A) Determination of CK2 enzymatic activity of the generated variants. The best performing variants as well as five random variants were selected for sanger sequencing and are highlighted accordingly. (B) Determination of CK2 enzymatic activity of WT, K198R, K198T and the previously best performing variant K198Q. Data are presented as mean ± SD (*n* = 6 biological replicates). Significance was calculated by one‐way ANOVA with WT set as control (*P* > 0.05 = n.s.). All variants were supplemented with 20‐fold excess of CK2β.

To investigate whether the high frequency of the K198R variant in patients could be attributed to codon usage or mutational bias, all possible single‐nucleotide substitutions of the K198 codon AAA, as found in human *CSNK2A1* (geneID: ENSG00000101266.20), were compiled and summarized in Table 3. It turned out that K198 may be replaced by six other amino acids due to a single‐nucleotide exchange. Notably, only N (asparagine) can appear by two different codons (AAT and AAC) resulting from a single amino acid exchange, yet a K198N variant has not yet been observed in any OCNDS patient. It is well known that transitions A↔G and C↔T are more common in humans compared to transversions, for example, A↔C or A↔T [64]. While the transition/transversion rate should be 0.5 as for each transition, there exists two possible transversions, the observed rate has been reported to be closer to 3 : 1 within human exome regions [65, 66]. Hence, the variants GAA (K198E), AGA (K198R) and AAG (K198K), resulting from transitions, are expected to occur more frequently as other variants. This is not the case for OCNDS patients, however, because for 82 cases as described, 81 times the K198R variant was detected and for one K198T was present. For comparison, variations of D156 that were occurring in ten OCNDS patients were analyzed. D156 is part of the active triad in the catalytic loop of CK2α and hence its variation leads to a loss of function. Despite the small sample size of ten patients, five out of seven amino acid exchanges resulting from a singular nucleotide exchange occurred in at least one patient, indicating that there is no such bias for a single exchange like it appeared for K198R.

**Table 3:** Possible mutations of Lys 198 (**A**) and Asp156 (**B**) due to single nucleotide exchange and their respective occurrence in patients with OCNDS.

A			K198X		B			D156X	
Codon			Resulting amino acid	Patients	Codon			Resulting amino acid	Patients
**A**	**A**	**A**	**Lys (K)**	**X**	**G**	**A**	**T**	**Asp (D)**	**X**
**C**	A	A	Gln (Q)	‐	**A**	A	T	Asn (N)	2
**G**	A	A	Glu (E)	‐	**C**	A	T	His (H)	1
**T**	A	A	STOP	‐	**T**	A	T	Tyr (Y)	2
A	**C**	A	Thr (T)	1	G	**C**	T	Ala (A)	‐
A	**G**	A	Arg (R)	81	G	**G**	T	Gly (G)	1
A	**T**	A	Ile (I)	‐	G	**T**	T	Val (V)	‐
A	A	**G**	Lys (K)	X	G	A	**G**	Asp (D)	X
A	A	**C**	Asn (N)	‐	G	A	**A**	Glu (E)	4
A	A	**T**			G	A	**C**		

Codons prevalent in the human genome for amino acids at position 198 and 156 are written in bold. Red letters indicate single nucleotide exchanges in comparison to the prevalent codons.

## Discussion

In this work, 42 CK2α variants associated with Okur‐Chung neurodevelopmental syndrome (OCNDS) were analyzed. It could be confirmed that—excluding variants known to be completely inactive—the majority of variants exhibited a reduced CK2 enzymatic activity. This is in accordance with an earlier study for a smaller panel of variants [43]. For these low‐activity variants, it appears reasonable at first sight that their pathogenic effect is at least partly due to a reduced phosphorylation of *in vivo* substrates. Indeed, brain extracts of mice heterozygously expressing the variant K198R have been shown to have an overall reduced CK2 enzymatic activity compared to WT [67]. The implications of such a reduced activity are difficult to predict because CK2 is involved in a multitude of pathways. While a full knockout of CK2α is known to be embryonically lethal in mice [12], patients with OCNDS are heterozygous and still have a functional allele, which might at least partly compensate for a lack of activity. In patient fibroblasts carrying the fully inactive variant D156E on one allele, multiple *in vivo* markers of CK2 enzymatic activity such as the phosphorylation of S129 in Akt were unaltered compared to WT fibroblasts [43]. These results indicated that the enzymatic activity of WT CK2α on one allele is sufficient to fulfill certain core functions in cells. It is known that the overall CK2α mRNA and protein levels as well as mRNA levels are unaltered both in heterozygous patient fibroblasts and heterozygous CK2α^WT/K198R^ mice [43, 67]. Yet, no information about the relative abundance of WT versus mutant CK2α was given in these studies. The observations as described above may be explained by an overexpression of WT compared to mutant CK2α within these cells. Mutation specific antibodies or advanced mass spectrometry approaches might help uncover this in the future.

In addition to their lack of activity, inactive CK2α variants could serve as a kind of competitive inhibitor with regard to WT CK2α when binding to protein complexes or substrates. A recent review highlighted protein complexes and modulators that may alter CK2 substrate specificity [68]. For instance, by forming a complex with CaMKII and GluN2B, CK2α is able to phosphorylate S1480 in GluN2B, resulting in the regulation of the subcellular localization of GluN2B [69, 70]. An inactive CK2α could bind the same complex but not lead to phosphorylation of S1480. In this and in previous studies, it has been shown that many of the variants associated with OCNDS have no impact on CK2α_2_β_2_ holoenzyme formation [44]. Hence, they could also be competing with WT for binding to CK2β.

With N16I, E180K, R195Q, and Y196C, variants have been identified in which CK2 activity was closer to WT activity after the addition of CK2β. This is likely due to their location in the N‐terminal segment and the activation segment of CK2α. For CK2α in its monomeric form, the binding of the N‐terminal segment to the activation segment is necessary for catalytic activity [71, 72]. Specifically, N16 and E180 have been described to be involved in this binding, explaining the strong effect of N16I and E180K on the activity of the α‐subunit [71]. When CK2α is bound to CK2β, the N‐terminal segment is no longer attached to the activation segment, and a possible effect of the mutation would be reduced.

The majority of variants associated with OCNDS showed a reduced CK2 enzymatic activity; however, there were variants which exhibited no altered enzymatic activity toward the canonical substrate peptide RRRDDDSDDD. These five variants R21Q, T127M, E264D, E282K, and R333* are located at positions that are not highly conserved throughout evolution. Hence, the absence of an effect on core functions of CK2α such as enzymatic activity is not surprising. The question arises, whether the mutations are misdiagnoses or whether there are other factors leading to pathogenity.

The variant R21Q was identified in an 8 ‐year‐old girl from Iran in 2022 [36]. The patient showed many of the symptoms associated with OCNDS such as developmental delay, hypotonia, behavioral issues, and sleep disorders. The additional hydrogen bond between Q21 and D25 as found in the crystal structure could increase the rigidity of the helix αA1 and reduce the flexibility of the N‐terminal segment. An increased rigidity is not in congruence with reduced thermostability as observed, as commonly high rigidity is rather associated with higher thermostability [73]. Nevertheless, as flexibility of the N‐terminal segment allows to stabilize CK2α by binding to the activation segment, a reduction of flexibility in this domain could in turn lead indeed to a reduced stability. This could have an effect on functionality as the melting point of 39.2 °C is close to physiological body temperature. Hence, there might be less active CK2α available in the body at a given time. As the N‐terminal segment, where R21Q is located, is close to the substrate binding site when CK2α is in its monomeric form, R21Q could also have an impact on the phosphorylation of class II substrates, which are only phosphorylated by the free catalytic subunit [74].

The variant T127M was found in a large‐scale screening of risk genes in patients with neurodevelopmental delay. Unfortunately, no further information about symptoms of the patient is available. It is furthermore unclear whether mutations in other disease‐causing genes exist for this patient that could help to explain the phenotype. As T127 is located on the surface of CK2α, it could be involved in the binding of substrates or other interaction partners and therefore have pathogenic *in vivo* effects. Further, the exchange to M127 could lead to affected solubility, as proposed earlier [38]. Parts of helix αD that T127 is a part of are involved in substrate recognition, namely at the P‐2 and P‐4 site [75]. The loss of a hydrogen bond between T127 and L124 could lead to a destabilization of helix αD. Hence, a destabilized helix αD could impact substrate recognition and, in consequence, lead to a reduced enzymatic activity. Alternatively, this could also lead to an altered substrate specificity. In addition, an effect on co‐substrate binding could also be possible because the ATP‐binding site is in direct proximity to helix αD. It remains open for what reason T127M‐mScar in cell lysates was not impacted with regard to catalytic activity. It cannot be excluded that the complex matrix in the cell lysates had a stabilizing effect that compensated for the structural destabilization.

The variant E264D was the most conservative mutation tested, because the replacement of a glutamic acid by an aspartic acid leads only to the loss of a single CH_2_ group in the side chain, while keeping all other chemical properties. As E264 is located on the surface of CK2α, the effect of such an altered side chain appears negligible at first sight. The crystal structure of variant E264D did not reveal any structural changes in the essential kinase domains, which is in congruence with its unaltered enzymatic activity. The existence of the same amino acid exchange in multiple other organisms as well as in human CK2α′ emphasizes that the core function of position 264 likely remains unaltered with the E264D exchange. E264D is one of multiple differences between CK2α and CK2α′ in the region surrounding the αGH1‐helix of CK2α/CK2α′. It is well known that CK2α and CK2α′ have distinct biological functions [63, 76]. However, little is known about the role of the αGH1‐helix. It appears plausible that this region could serve as a binding area for proteins differentiating between CK2α and CK2α′. As a result, E264D might influence this differentiation and therefore potentially behave more like CK2α′. However, as of now this remains speculative and requires further studies, for example, by interactome or phosphoproteome analysis.

The variant E282K could not be crystallized because E282 is necessary for crystal packing. However, as E282 is also located on the surface of CK2α, it is likely involved in either binding of other substrates or—also plausibly—for an increasing solubility. An exchange of E282 to K is leading to a charge change and could have an effect on both of these. As no patient data are available for E282K, it cannot be excluded that the variant is benign and other factors in the patient were involved in the observed phenotype. A recent study by Bagatelas *et al*. [41] highlighted that variants in the N‐terminal segment and in the C‐terminal interdomain region show less severe phenotypes. Although the variants R21Q, E264D, and E282K have not been part of their study, the low *in vitro* effect of these amino acid exchanges as observed in our study is in accordance with these results.

The variant R333* still fulfills key functions of CK2α *in vitro*. As described above, the role of the C terminus in the function of CK2α is still unclear. It has been shown, however, that the C terminus of CK2α is necessary for the binding of certain proteins such as the peptidyl‐prolyl isomerase Pin1 [77]. Therefore, an impaired interactome could be a reason for the pathogenicity of R333*.

The variants R21Q, T127M, E264D, E282K, and R333* would be interesting candidates for animal models to elucidate whether these variants are in fact leading to a neuropathological phenotype or represent rather benign variants. Additionally, methods such as photo‐crosslinking [77], or phosphoproteome analysis [42] in lysates of cells expressing the variants or supplied with the variants could be used to elucidate effects on a cellular level. Additionally, this could improve the understanding of the function of CK2α domains such as the αGH1‐helix or the C‐terminal region.

All enzymatic activities in this study have been determined with the canonical CK2 substrate peptide RRRDDDSDDD. It contains the consensus CK2 phosphorylation motif [4], which has been used throughout numerous studies before [52, 53, 54, 55], and the activities determined can serve as a first indication of a reduced overall catalytic function. However, in case an amino acid exchange in CK2α leads to an alteration of the substrate spectrum *in vivo*, this cannot be detected by these means. In addition, while the enzymatic activities as measured are useful as a first impression, a variant that has an impact on the binding of substrates or recruiter proteins that similarly lead to a reduced phosphorylation of physiological substrates will not be sensed.

The results of the site saturation mutagenesis of Lys198 showcased that while all other substitutions at this position might lead to a similar loss of catalytic activity, only K198R is consistently observed in patients. In mice, the embryonical lethality of heterozygous CK2α^WT/K198R^ mice is about 50% [67]. In comparison, a heterozygous knockout of CK2α did not show increased embryonical lethality, despite a 32% reduction of CK2 activity in embryos [12]. This suggests that the pathogenicity of OCNDS‐associated variants could be influenced by factors beyond the loss of activity. In the case of K198R, the conservative exchange of a lysine to an arginine has been documented to have an effect on the substrate specificity of CK2α [42, 44]. Such changes might lead to altered phosphorylation events and, hence, potentially to a toxic effect of the mutation. K198X variants with less conservative amino acid exchanges might lead to a stronger impact on substrate specificity and could therefore be even more pathogenic compared to K198R. As a result, a more pathogenic K198X variant could lead to higher lethality at an embryonic stage or early development, leading to a survivorship bias of K198R. Incidentally, while for K198R adult patients exist, the only patient with another variant of K198 (K198T) was at the age of two months at the time of publication [37]. Hence, there is no information on the long‐term effects and survivability of K198T or other potential constraints. Another explanation could be that individuals carrying K198X variants other than K198R or K198T do not exhibit a strong phenotype and therefore were not discovered by whole exome sequencing, in particular of OCNDS patients. It could be speculated that, for example, the substrate specificity of these variants is less impacted in comparison to K198R and as a result, the variants would exhibit a reduced catalytic activity, but not gain a potential toxic function. This could lead to a less severe phenotype in individuals compared to K198R.

In conclusion, this study shows that while reduced enzymatic activity is common among OCNDS‐associated CK2α variants, it appears not to be the sole determinant of pathogenicity. Other mechanisms such as altered stability, substrate specificity, or protein–protein interactions must be considered to fully understand the functional impact and the genotype–phenotype relationships of individual mutations.

## Material and methods

### Cloning and site‐directed mutagenesis

The gene coding for CK2α^1‐335^‐mScarlet (CK2α‐mScar) was embedded in a pET‐11d vector (69 439 Novagen, Merck). The resulting fusion proteins contained an N‐terminal (His)_6_‐tag and a flexible linker (SGGGG) between CK2α^1–335^ and the C‐terminally fused mScar.

The gene coding for CK2α^1–335^ without fused mScar was embedded in a pET‐28a (+) plasmid (69 864 Novagen, Biocat) and thus resulted in a protein with an N‐terminal (His)_6_‐tag.

Site‐directed mutagenesis was used to generate mutant CK2α^1–335^ or CK2α^1‐335^‐mScar variants.

### Selection of OCNDS‐related CK2α variants

The focus was set on variants that have not been studied before in the study by Dominguez *et al*. [43]. As the C‐terminally truncated variant CK2α^1–326^ has been shown to be inactive before, nonsense and frameshift mutations before position 326 were not considered, because they were suspected to have no enzymatic activity [78]. Variants affecting residues previously shown to be essential for catalytic activity (e.g., Ser51, Asp156, Lys158, Asn161, Asp175) were excluded, as these mutations were expected to result in complete loss of function as well [8]. Additionally, because for studies on enzymatic activity in most cases a truncated CK2α^1–335^ is used [15, 44, 55, 79, 80], missense variants after position 335 were also excluded. For better comprehensibility, CK2α^1–335^ will be referred to as CK2α or wild‐type (WT) in the following. Table 1 gives the selected variants with their location in the different domains of CK2α. In addition to the variants published before, variants described in the registry of the CSNK2A1‐foundation (https://www.csnk2a1foundation.org/registry‐dashboard retrieved 20th of May, 2025) have been included.

### Preparation of proteins used in this work

The purification of CK2α^1‐335^‐variants, CK2α^1‐335^‐mScar and CK2β^1–193^ was performed as described previously [45]. The preparation of CK2α^1‐335^‐mScar variant cell lysates as well as the quantification of CK2α^1‐335^‐mScar was performed as in a recently published protocol [45].

### Determination of CK2 activity by capillary electrophoresis

CK2 activity measurements were based on a capillary electrophoresis‐based activity assay [56]. Modifications were applied as described previously [45]. Briefly, the CK2α concentration in *Escherichia coli* cell lysates was determined by measuring the red fluorescence of the fused mScar using a Tecan M200 plate reader (excitation: 561 nm, emission: 610 nm) and using an external calibration of purified CK2α‐mScar. Lysates with similar CK2α‐mScar concentrations were selected and 5 μL of the respective lysate were supplemented to 95 μL kinase buffer (150 mm NaCl, 25 mm MgCl_2_, 25 mm Tris/HCl, pH 7.5), and the fluorescence was measured again. This resulted in reaction vessels containing final concentrations of around 10 nm CK2α‐mScar. After incubation for 10 min at 30 °C, the reactions were started by addition of 100 μL assay buffer (150 mm NaCl, 25 mm MgCl_2_, 600 μm substrate peptide RRRDDDSDDD, 600 μm ATP, 25 mm Tris/HCl, pH 7.5). The reaction was stopped by transferring samples into 25 μL of EDTA (0.5 M, pH 8.5) after 90 min (CK2α) or 30 min (CK2α + CK2β).

### Determination of dissociation constants within cell lysates

Dissociation constants (*K*_D_‐values) were determined in *Escherichia coli* cell lysates as described previously [45]. The method was based on the dose‐dependent activity increase of CK2α activity after addition of CK2β. The resulting activity curves can be interpreted as binding isotherms and therefore be used to fit a binding model for *K*_D_‐determination [81]. The probability distribution of the accurate *K*_D_‐values was determined using a Monte Carlo approach implemented in a custom Python program employing the publicly available *scipy* package for curve fitting by nonlinear regression and kernel density estimation and the *numpy* package for random sampling and integration [82, 83]. The Monte Carlo simulation resulted in an array of 50 000 *K*_D_,det‐values. The 95% confidence interval was determined from the array of *K*_*D*,det_ from the Monte Carlo Simulation with the 2.5th percentile as the lower bound and the 97.5th percentile as the upper bound.

### Determination of thermostability

Thermostability was determined using differential scanning fluorimetry (DSF) as described previously [84]. Briefly, 1 μm of the respective CK2α‐variant with or without 2 μm of CK2β were solved in PBS and supplemented with 20 × SYPRO Orange (Sigma Aldrich, St. Louis, USA). Samples were incubated on ice for 10 min and then inserted into the Rotor‐Gene Q 2 Plex HRM (Qiagen, Hilden, Germany). The sample temperature was set to 30 °C and then continuously heated by 0.5 °C/5 s until a temperature of 95 °C was reached. During this process, the samples were excited at 470 ± 10 nm and the emission of SYPRO Orange was measured at 610 ± 5 nm. Melting points were determined as the extreme points of the first derivative of the Fluorescence signal (dF/dT(T)) by using the Rotor‐Gene Q software (Qiagen, Hilden, Germany).

### Crystallization and determination of crystal structures

To crystallize the respective mutant, we followed the technique used to get crystals of the CK2α variant K198R [44]. The reservoir solution was 200 mm lithium sulphate, 100 mm Bis‐TRIS, pH 6.5, 35% PEG 3350, an optimization from the condition G3 of the INDEX screen (Hampton research, Aliso Viejo, CA, USA). A 5 mg·mL^−1^ stock solution of the respective variant in standard buffer (500 mm NaCl, 25 mm TRIS/HCl, pH 8.5) was mixed 2 : 1 (T127M, E164D) or 3 : 1 (R21Q) with the reservoir solution. To get crystals of mutant R21Q with adequate diffraction properties, we optimized initial crystals by microseeding. Fully grown crystals were harvested, cryo‐protected with 30% (v/v) ethylene glycol in reservoir solution, and flash‐frozen in liquid nitrogen. Data for the R21Q structure were collected on ID30A‐3 (MASSIF‐3, ESRF) and for E264D and T127M on ID30B (ESRF). We could not crystallize the mutant E282K, likely due to crystal contacts in which E282 is involved. Also, extended screening did not yield suitable crystals; therefore, we could not provide a structure of this mutant.

Data were processed with the autoPROC toolbox [85]. autoPROC executes XDS [86], aimless, and pointless [87] from the CCP4 suite [88] and Staraniso [89]. The phases were solved by molecular replacement in Phaser [90] using PDB structure 2PVR [91] as the input model. Iterative cycles of refinement in Coot [92] and phenix.refine [93] followed. Table 2 provides detailed X‐ray diffraction data for the generated structure. The respective PDB files can be accessed under the codes 9RCY (R21Q), 9RCX (T127M), and 9RFN (E264D).

### Site‐saturated mutagenesis

Libraries containing the genes coding for CK2α^K198X^‐mScar were constructed by site saturation mutagenesis as described before [94]. Primers containing a degenerate codon (NNK) instead of the codon coding for Lys198 were ordered from Sigma‐Aldrich (St. Louis, MO, USA). They were used to insert the degenerate codon into a plasmid containing the gene coding for CK2α‐mScar. Template DNA was removed by DpnI treatment (2 h, 37 h). The PCR‐product was used to transform E*scherichia coli* DH5α. After incubation for 1 h at 37 °C in SOC Medium, cells were plated onto an agar plate carrying 50 μg·L^−1^ Carbenicillin. After incubation for 16 h at 37 °C, colonies were pooled of the plate by scraping using a rubber policeman and 5 mL PBS. Plasmid isolation was performed and the product was confirmed by Sanger sequencing. The quality of the received library was determined using the Q_pool_ method [95]. In the Q_pool_ method the distribution of amino bases at each position of the NNK codon received from a standard Sanger sequencing result is compared to an ideal distribution of bases (1 : 1 : 1 : 1 for N, 1 : 1 for K). The resulting Q_pool_‐value varies between 0 (no randomization) and 1 (perfect randomization). The resulting value for the CK2α^K198X^‐library was determined as Q_pool,K198X_ = 0.76. According to Sullivan *et al*. [95] a Qpool > 0.7 may lead to a representative randomization, indicating that the library was sufficiently randomized.

## Author contributions

AG designed and performed the studies, analyzed data, and wrote the manuscript. CW solved the crystal structures. JJ designed the project, revised and edited the manuscript, and acquired funding, KN designed the project and acquired funding. All authors read and approved the manuscript.

## Conflict of interest

The authors declare no conflict of interest.

## Supporting information


**Fig. S1.** Multiple sequence alignment.

**Fig. S2.** Amino acid conversation scores.

**Table S1.** Activity of CK2α variants with and without addition of excess of CK2β.

## Data Availability

The data that support the findings of this study are available from the corresponding author (joachim.jose@uni-muenster.de) upon request. The atomic coordinates and structure factor amplitudes of the three CK2α mutant crystal structures can be downloaded from the Protein Data Bank (PDB) via the accession codes 9RCY (CK2α^R21G^), 9RCX (CK2α^T127M^) and 9RFN (CK2α^E264D^). The raw X‐ray diffraction data are available at the ESRF via https://doi.org/10.15151/ESRF‐ES‐2037820738 for the CK2α^R21G^ structure and via https://doi.org/10.15151/ESRF‐ES‐1975656184 for the CK2α^E264D^ and the CK2α^T127M^ structures.
