# Supplementary material for: Functional characterization of 42 CK2α de novo variants associated with Okur‐Chung neurodevelopmental syndrome
Source: FEBS J. 2026 Apr 13;293(19):5759–79. doi: 10.1111/febs.70538 (PMC13628543; doi:10.1111/febs.70538)
Supplement: Supplementary file 1 [file FEBS-293-5759-s003.pdf]

Supplementary Table S1: Activity of CK2α variants with and without addition of excess of CK2β.

Variant	K_{cat} [min^{-1}]		Relative activity to WT		Fold increase of activity [$CK2\alpha_2\beta_2$ / CK2α]
	CK2α	$CK2\alpha_2\beta_2$	CK2α	$CK2\alpha_2\beta_2$	
**WT**	57	497	100%	100%	8.8
**N16I**	22	299	38%	60%	13.9
**R21Q**	47	507	84%	102%	10.7
**E27K**	34	355	60%	72%	10.4
**Y39C**	0	0	0%	0%	-
**Y39S**	0	0	0%	0%	-
**G46V**	0	0	0%	0%	-
**G48S**	5	22	9%	4%	4.1
**Y50N**	7	101	12%	20%	15.2
**E52K**	31	174	55%	35%	5.6
**V53L**	2	57	3%	12%	35.8
**V66L**	3	23	6%	5%	7.0
**L70P**	2	43	3%	9%	26.5
**L70R**	9	80	17%	16%	8.5
**V73E**	14	77	26%	16%	5.4
**T127M**	55	488	98%	98%	8.8
**C147Y**	0	0	5%	0%	-
**M153R**	0	0	1%	0%	-
**I174M**	4	78	8%	16%	17.8
**G177S**	2	18	4%	4%	8.7
**L178W**	3	11	5%	2%	4.1
**E180K**	9	134	16%	27%	14.5
**R191P**	1	19	2%	4%	14.2
**A193P**	0	0	2%	0%	-
**S194F**	0	0	0%	0%	-
**S194P**	0	0	0%	0%	-
**R195Q**	19	249	33%	50%	8.0
**R195P**	15	124	27%	25%	13.2
**Y196C**	15	282	27%	57%	18.5
**F197I**	1	48	2%	10%	45.4
**K198R**	4	17	7%	3%	4.2
**K198T**	1	6	1%	1%	7.9
**G199D**	0	0	0%	0%	-
**D214V**	0	0	0%	0%	-
**S217N**	6	76	10%	15%	13.1
**A223E**	0	0	0%	0%	-
**P231R**	13	88	23%	18%	6.8
**Q241E**	4	13	8%	3%	2.9
**E264D**	56	587	99%	118%	10.5
**E282K**	51	417	91%	84%	8.1
**R312W**	0	0	0%	0%	-
**R312Q**	0	0	0%	0%	-
**R333***	55	523	97%	105%	9.6
